# Rate and risk factors for shunt revision in pediatric patients with hydrocephalus during a 17-year study period: a retrospective, population-based study

**DOI:** 10.1007/s00381-025-06992-1

**Published:** 2025-10-18

**Authors:** Juho Välimaa, Terhi J. Huuskonen, Joona Tervonen, Ville Leinonen, Susanna Rantala

**Affiliations:** Neurosurgery KUH NeuroCenter, Faculty of Health Science, School of Medicine, Institute of Clinical Medicine, Kuopio University Hospital, University of Eastern Finland, Kuopio, Finland

**Keywords:** Hydrocephalus, Pediatric, Shunt, Revision

## Abstract

**Purpose:**

We studied the surgical outcomes of pediatric hydrocephalus (HC) patients requiring primary cerebrospinal fluid (CSF) diversion surgery with ventriculoperitoneal shunt (VPS) in a retrospective population-based study.

**Methods:**

The medical records of 92 patients under 16 years of age with HC requiring primary VPS were reviewed. Patients with Strata adjustable valve were studied in their own cohort. The rate and the risk factors for shunt revision were analyzed.

**Results:**

We found 92 patients treated with primary VPS. Strata adjustable valve shunt was used in 43 patients. The mean age at the time of primary shunt insertion was 3.2 years (SD 4.7 years). The mean follow-up time for shunted patients was 5.9 years (SD 6.8 years), and 5.3 years (SD 5.3 years) for the Strata valve subgroup. The most common etiologies of HC were tumors (29.3%), congenital defects (CD) (21.7%), and intraventricular hemorrhage (IVH) (22.8%) in shunted patients. During the follow-up period, 54.3% of all shunted patients and 48.8% of Strata valve shunted patients underwent at least one revision surgery. The mean time to revision surgery was 1.0 years. Revision rates in the CD group (70%) were higher compared to the tumor group (37%) (*P* = 0.044). Patients younger than 6 months had higher shunt revision rates than older patients (*P* = 0.036).

**Conclusions:**

Out of the 92 shunted patients, 54.3% underwent revision surgery, with most revisions occurring within the first year after initial VPS placement. Patient characteristics, such as congenital defects and age under 6 months, were associated with a higher risk for shunt revision. The use of the Strata adjustable valve did not have a statistically significant impact on shunt revision rates.

## Introduction

Hydrocephalus (HC) is caused by a disruption in the physiological flow of cerebrospinal fluid (CSF), resulting in the expansion of the cerebral ventricles and a possible increase in intracranial pressure [18]. HC is the most common pediatric brain disorder, leading to a variety of debilitating complications, such as cognitive dysfunction, blindness, or even death if not promptly treated. Because of its high prevalence in the pediatric population, neurosurgeons are frequently faced with HC-related surgeries. Previously published meta-analyses indicate a pediatric HC prevalence of 88/100,000 and an incidence varying globally between 68/100,000 and 316/100,000 births, with significantly higher rates seen in low-income countries [10, 17]. Many maternal and infantile risk factors for the development of childhood onset HC have been identified in previous studies. Infantile risk factors, such as prematurity, male sex, first- or second-degree relatives with HC, or being part of a set of multiples, have shown limited evidence of leading to congenital HC [40]. In addition, maternal factors such as obesity, type 1 or type 2 diabetes, thyroid disease, pre-eclampsia, lack of adequate multivitamin supplementation, and the use of antidepressants, opioids, tribenoside, metronidazole, or proton pump inhibitors during the first trimester are considered to increase the child’s risk of congenital HC [40]. In the literature, the most common etiologies of HC in children are intraventricular hemorrhage (IVH), congenital defects (CD), tumors, and aqueductal stenosis (AqS) [3, 8, 23, 28, 29, 36, 38].

Surgical procedures have proven to be a critical component in the treatment of HC and the prevention of associated complications. Ventriculoperitoneal shunt (VPS) remains the most widely used method for CSF diversion, although neuroendoscopic surgeries, such as endoscopic third ventriculostomy (ETV), have gained wider acceptance in recent decades [42, 44]. VPS has been shown to be prone to complications related to the catheter (e.g., migration, obstruction, or misplacement), the valve (e.g., obstruction or overdrainage), or infection, often leading to shunt revision surgery [8, 35, 36]. This susceptibility of VPSs to complications is reflected in high revision rates ranging from 18 to 85% according to the literature [2–4, 6, 8, 19, 22, 24, 25, 28, 29, 31, 34–36, 39, 41], with various factors associated with increased risk of shunt revision, as well as the length of follow-up. These risk factors include, for example, prematurity, the etiology of HC, young age at primary surgery, and male sex [4, 22, 28, 35, 36].

Adjustable shunt valves have been developed to decrease the need for shunt revisions since the valve setting can be changed without surgical intervention in order to treat over- or underdrainage. Several studies have demonstrated that the use of adjustable shunt valves reduces the rate of revisions and complications related to over- or underdrainage compared to fixed-pressure valves, particularly in pediatric patients with hydrocephalus [20, 24, 43]. Furthermore, improvements in clinical status have been reported following valve adjustments [45]. However, no significant differences have been observed in catheter-related complications or infection rates [20, 43]. Conversely, the overall evidence remains inconclusive, and some studies have failed to demonstrate a difference in revision rates between adjustable and fixed-pressure valves [1, 11, 15, 25, 30, 36]. While valve adjustment is straightforward and non-invasive, potential complications specific to adjustable valves include inadvertent alterations in valve setting due to exposure to magnetic fields or postural variations, as well as inaccurate opening pressure measurements [9, 16, 43, 45].

Previously, we published a study on pediatric shunt patients [36]. In the time period from 2003 to 2013, we found that age under 6 months, IVH, and CD were associated with an increased risk for shunt revision. In the present study, we included all the pediatric patients treated with primary shunt from 2003 to 2019. Our shunt treatment policy changed after 2009, from which we have predominantly used Strata adjustable valves. Our aim was to further investigate the long-term survival of pediatric shunts by comparing the more recent cohort of patients treated with adjustable valves to the previous cohort in a population-based setting and to assess rates and risk factors for revision surgery.

## Materials and methods

### Study populations

The present study is a population-based retrospective study of all pediatric patients under 16 years of age who underwent their primary CSF diversion surgery with VPS for the treatment of HC. The study cohort includes all pediatric patients from the Kuopio University Hospital (KUH) catchment area who underwent primary shunt surgery between January 1, 2003, and December 31, 2019. The Department of Neurosurgery at KUH has been the sole provider of full-time elective and acute neurosurgical care for Central and Eastern Finland since 1977, with a catchment population of 797,234 inhabitants in 2021. The study was approved by the Ethics Committee of the KUH (approval number 5252659).

### Clinical and surgical variables

Analyses for all pediatric patients with HC were performed using the following variables:

1. Patient-related variables: gender, age at the initial VPS surgery, birth weight, and possible infections (respiratory infections or meningitis) prior to shunt surgery.
2. Etiology of HC: IVH, tumor, congenital defect, ICH, aqueductal stenosis, meningitis, bilateral stenosis of the sigmoid sinus–jugular vein complex, unspecified HC, and trauma.
3. Surgery-related variables: ventricular reservoir (Rickham capsule) or external ventricular drain (EVD) placement prior to the primary shunt surgery, shunt valve type, site of ventricular catheter placement, ventricular catheter type, and number of valve adjustments performed. Valve adjustments made due to MRI imaging were not included in the study. Fixed-pressure shunt valves were used in the early years, after which adjustable valves (Codman, later Strata II/NSC) were gradually adopted. Since 2009, only Strata adjustable valves have been implanted, reflecting evolving institutional practice and changes in clinical preference over time.
4. Surgical outcome variables: time to the first revision, cause, and number of revisions.
5. Follow-up data until September 2024 and all causes of death.

### Follow-up

Follow-up of the study cohort was performed at the Department of Neurosurgery at KUH. Suspected shunt malfunction and other complications were treated exclusively at KUH. Appropriate imaging studies were performed at KUH or at the central hospitals in the catchment area of KUH. Adjustments to the settings of adjustable valves were made based on clinical observations during routine follow-up visits or during acute evaluations for suspected shunt-related malfunction. Decisions regarding valve adjustments were made on a case-by-case basis, guided by the patient’s clinical presentation and findings on head ultrasonography or MRI.

### Literature review

**Fig. 1:**
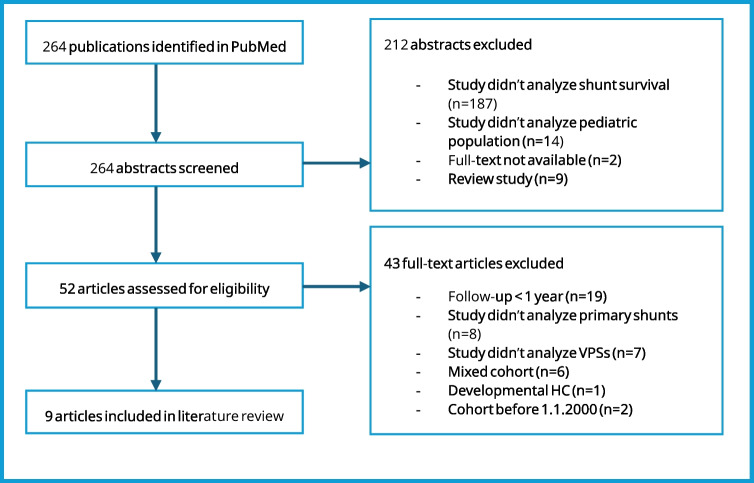
Flow chart of the literature search and the selection of previous relevant clinical studies on shunt surgery

The PubMed search was performed using the following key words: “pediatric,” “hydrocephalus,” “shunt,” and “revision” from January 1, 2017, to September 30, 2024. In our previous article [36], we performed a literature review for pediatric shunt survival from January 2000 to December 2016. Studies with no full-text version available were excluded. Abstracts were screened for studies that analyzed shunt survival with a minimum of 1 year of follow-up. From the literature search, 52 full-text articles were evaluated, and nine relevant cohorts were identified. The flow chart in Fig. 1 shows the exclusion criteria. The summary of relevant cohorts is shown in Table 1*.*

**Table 1:** Characteristic of previous relevant pediatric shunt cohorts

Series	Country	Time period	Number of patients	Mean age	Mean follow-up	Most common etiologies	Revision rate	Risk factors
Tervonen et al., 2017 [36]	Finland	2003–2013	80	3.2 years	3.3 years	Tumor (27.5%), congenital HC (22.5%), IVH (18.8%)	51%	Age < 6 months, IVH, congenital HC
Anderson et al., 2019 [2]	UK	2010–2014	321 (126 primary shunts)	2 years	3.5 years	Vascular (27.2%), dysraphism (26.6%), congenital malformation (22.2%)	26% (1-year failure rate)	Lack of Consultant Involvement in operation, single-surgeon operation^b^
Kommer et al., 2019 [19]	UK	2012–2015	61	3.1 years	38.8 months	Tumor (27.9%), congenital HC (21.3%), IVH (13.1%)	47.5%	N/A
Rymarczuk et al., 2020 [31]	USA	2000–2013	459	2.3 years	5.9 years	GMH (28%), neoplasm (17%), spinal dysraphism (16%)	54%^c^	N/A
Azzam et al., 2021 [4]	Indonesia	2018–2019	142	37 months	1 year	N/A	18%	Age < 6 months
Mansoor et al., 2021 [22]	Norway	2008–2017	81	8 months^a^	73 months^a^	Congenital HC (23.5%), tumor (17.3%), AC (13.6%)	58%	Congenital HC, MMC, young age
Prajapati et al., 2022 [28]	India	2016–2019	152	53 months	1 year	AqS (43.4%), TBM (20.4%), MMC (16.5%)	33%	N/A
Wendling-Keim et al., 2023 [41]	Germany	N/A	81	N/A	226 months	ICH (49.4%), MMC (23.5%), congenital HC (19.8%)	72.8%	Gravitational valve
Chiarelli et al., 2024 [6]	USA	2003–2018	76	33 days	47 months^a^	IVH (54%), open NTD (29%), AqS (13%)	51%	N/A

*HC*, hydrocephalus; *IVH*, intraventricular hemorrhage; *GMH*, germinal matrix hemorrhage; *AC*, arachnoid cyst; *MMC*, myelomeningocele; *AqS*, aqueductal stenosis; *TBM*, tubercular meningitis; *ICH*, intracerebral hemorrhage; *NTD*, neural tube defect; *N/A*, not applicable

^a^Reported as median; ^b^evaluated at 30 days after shunt; ^c^VP shunts

### Statistical analyses and artwork

**Fig. 2:**
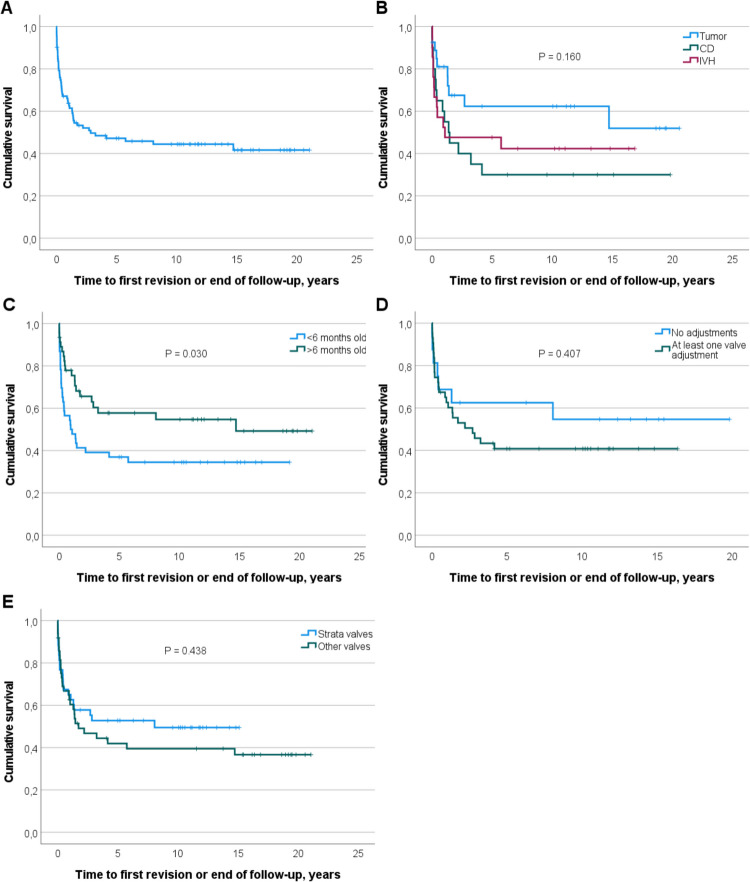
Kaplan–Meier survival functions. **A** Overall shunt survival rate. **B** The three most common etiologies of HC compared with each other: tumor, congenital (CD), and IVH. **C** Under 6-month-old patients at the primary shunt operation compared with older patients. **D** Adjustable pressure valves with at least one valve adjustment compared with adjustable pressure valves that remained unadjusted. **E** Strata adjustable valves compared with other valve types

Means and standard deviations (SDs) were used to present continuous variables, and frequencies and percentages were used to present categorical and dichotomous variables. Independent samples *t*-test for continuous variables and Pearson’s chi-squared test for discrete variables were used for group comparisons between patients who underwent shunt revision. In categorical variables with small sample sizes, Fisher’s exact test was used. Overall survival of shunt, i.e., time to shunt revision or end of the follow-up period, was estimated by using the Kaplan–Meier method. The log-rank test was used to compare revision-free shunt survival between patients with different HC etiologies, different numbers of adjustable valve adjustments, different age groups, and different valve types. Multivariate logistic and Cox regression analyses were performed to identify the factors with an independent effect on shunt survival. Analyses were performed using IBM SPSS Statistics for Windows version 27.0 (IBM Corp., Armonk, NY, USA). Differences were considered statistically significant when the *P* value was < 0.05. The confidence interval was 95%. Microsoft Office 365 was used to create Fig. 1. Figure 2 was created with IBM SPSS.

## Results

### Patient characteristics

In this study, we found a total of 92 primary ventriculoperitoneal shunt procedures from January 2003 to December 2019. A Strata adjustable valve was used in the primary shunt implantation with 43 (46.7%) patients. The majority of patients were male (*n* = 58, 63%), and 50% (*n* = 46) were older than 6 months at the time of initial surgery. The mean age at the time of primary VPS surgery was 3.2 years (SD 4.7 years). Birth weight was recorded in 69 (75%) patients, and overall, 9 (9.8%) children weighed under 1500 g at birth. The mean birth weight was 3031 g (SD 1017 g). All patient characteristics are presented in Table 2*.*

**Table 2:** Characteristics of pediatric patients with primary shunt

Characteristics		All patients with shunt	Patients with revised shunt	P value (all shunt revisions)	Patients with Strata valve	Patients with revised Strata valve	P value (Strata)
Total		92	50 (54.3%)		43 (46.7%)	21 (48.8%)	
Male patients		58 (63%)	31 (53.5%)	0.821	30 (69.7%)	13 (43.3%)	0.276
Age, years, mean (SD)		3.2 (4.7 years)	2.4 (4.4 years)	0.076	2.8 (4.7 years)	2.1 (4.5 years)	0.387
Age groups	< 6 months	46 (50%)	30 (65.2%)	0.036*	23 (53.5%)	12 (52.2%)	0.639
	≥ 6 months	46 (50%)	20 (43.5%)		20 (46.5%)	9 (45%)	
Birth weight, g, mean (SD)		3031 (1017)^a^	2944 (1103)^b^	0.395	2892(1095)^c^	2668 (1146)^d^	0.267
Birth weight < 1500 g		9 (9.8%)	7 (77.7%)	0.299	6 (14%)	4 (66.7%)	0.402
Reason for primary shunt				0.118			0.181
	Tumor	27 (29.3%)	10 (37%)		11 (25.6%)	4 (36.4%)	
	IVH	21 (22.8%)	12 (57.1%)		14 (32.6%)	7 (50%)	
	Congenital	20 (21.7%)	14 (70%)		6 (14%)	2 (33.3%)	
	AqS	10 (10.9%)	7 (70%)		4 (9.3%)	4 (100%)	
	HC unspecified	7 (7.6%)	5 (71.4%)		3 (7%)	2 (66.7%)	
	Trauma	3 (3.3%)	2 (66.7%)		2 (4.7%)	2 (100%)	
	ICH	1 (1.1%)	1 (100%)		1 (2.3%)	0	
	Meningitis	2 (2.2%)	0		1 (2.3%)	0	
	SSS	1 (1.1%)	0		1 (2.3%)	0	
Rickham capsule before shunt		14 (15.2%)	9 (64.3%)	0.418	10 (23.3%)	6 (60%)	0.488
EVD before shunt		14 (15.2%)	6 (42.9%)	0.349	8 (18.6%)	2 (25%)	0.240
Infection before shunt		11 (12%)	7 (63.6%)	0.510	7 (16.3%)	3 (42.9%)	1.000
Ventricular catheter type				0.906			0.876
	Silicone	67 (72.8%)	37 (55.2%)		22 (51.2%)	10 (45,5%)	
	Bactiseal	23 (25%)	12 (52.2%)		19 (44.2%)	10 (52,6%)	
	Silverline	2 (2.2%)	1 (50%)		2 (4.7%)	1 (50%)	
Shunt valve type				0.362			
	Strata, adjustable	43 (46.7%)	21 (48.8%)		43 (100%)	21 (48.8%)	
	Strata II	37 (40.2%)	18 (48.6%)		37 (86%)	18 (48.6%)	
	Strata NSC	6 (6.5%)	3 (50%)		6 (14%)	3 (50%)	
	Medtronic 1.5	21 (22.8%)	10 (47.6%)		0	0	
	Medtronic 1.0	5 (5.4%)	4 (80%)		0	0	
	Codman, adjustable	15 (16.3%)	10 (66.7%)		0	0	
	Codman Medium	5 (5.4%)	4 (80%)		0	0	
	Codman Micro	1 (1.1%)	1 (100%)		0	0	
	paediGAV	1 (1.1%)	0		0	0	
	GAV 5/30	1 (1.1%)	0		0	0	
Primary adjustable pressure valve		58 (63%)	31 (53.4%)	0.821	43 (100%)	21 (48.8%)	
At least one valve adjustment		43 (72.9%)	25 (58.1%)	0.324	31 (72.1%)	16 (51.6%)	0.558

Values are reported as numbers (percents) except for age and weight, which are reported as means (SDs). *P* values are calculated by independent samples *t*-test, Pearson’s chi-squared test, or Fisher’s exact test as appropriate

*IVH*, intraventricular hemorrhage; *AqS*, aqueductal stenosis; *HC*, hydrocephalus; *ICH*, intracranial hemorrhage; *SSS*, stenosis of sigmoid sinus–jugular vein complex; *EVD*, external ventricular drain; *VP*, ventriculoperitoneal

Statistically significant *P* values are marked by an asterisk symbol (*)

^a^In 69 patients; ^b^in 41 patients; ^c^in 34 patients; ^d^in 16 patients

The most common etiologies for primary shunt surgery were tumors (*n* = 27; 29.3%), IVH (*n* = 21; 22.8%), and CD (*n* = 20; 21.7%). In the tumor group, the most common tumors were medulloblastoma (*n* = 7; 25.9%), pilocytic astrocytoma (*n* = 4; 14.8%), glioma (*n* = 3; 11.1%), and plexus papilloma (*n* = 2; 7.4%). Other tumors observed were primitive neuroectodermal tumor (PNET) (*n* = 2; 7.4%), oligoastrocytoma (*n* = 2; 7.4%), subependymal giant cell astrocytoma (*n* = 2; 7.4%), ependymoma (*n* = 2; 7.4%), metastasis of retinoblastoma (*n* = 1; 3.7%), cavernous hemangioma (*n* = 1; 3.7%), and germinoma (*n* = 1; 3.7%). In the CD group, the most common etiology was myelomeningocele (*n* = 9; 45%). Other observed CDs were schizencephaly (*n* = 2; 10%), porencephaly (*n* = 2; 10%), hydranencephaly (*n* = 2; 10%), Dandy-Walker malformation (*n* = 1; 5%), encephalocele (*n* = 1; 5%), Aicardi syndrome (*n* = 1; 5%), holoprosencephaly (*n* = 1; 5%), and cloverleaf skull syndrome (*n* = 1; 5%). Other etiologies for primary CSF diversion surgery included AqS (*n* = 10; 10.9%), unspecified hydrocephalus (*n* = 7; 7.6%), trauma (*n* = 3; 3.3%), meningitis (*n* = 2; 2.2%), ICH (*n* = 1; 1.1%), and bilateral stenosis of the sigmoid sinus–jugular vein complex (*n* = 1; 1.1%).

All implanted shunts were ventriculoperitoneal. The Rickham capsule was implanted in 15.2% (*n* = 14) of patients prior to VPS insertion. IVH was the leading cause of Rickham capsule insertion in 12 (85.7%) patients. EVD was used in 15.2% (*n* = 14) of shunted patients. EVD was required due to HC caused by tumor in seven (50%) patients and in three (21.4%) patients with hemorrhage. The infection rate was 8.7% (*n* = 8) (Table 3).

**Table 3:** Causes for shunt revision

		**Number of revised shunts**	**Percentage of revised shunts**	**Percentage of all shunts**	**Number of revised Strata valves**	**Percentage of revised Strata valves**	**Percentage of all Strata valves**
Underdrainage		29	58.0%	31.5%	10	47.6%	23.3%
	Valve obstruction	5	10.0%	5.4%	1	4.8%	2.3%
	Valve failure for unknown reason	3	6.0%	3.3%	0	0%	0%
	Peritoneal catheter obstruction	2	4.0%	2.2%	0	0%	0%
	Ventricular catheter obstruction	11	22.0%	12.0%	6	28.6%	14.0%
	Ventricular catheter malposition	5	10.0%	5.4%	1	4.8%	2.3%
	Peritoneal catheter malposition	3	6.0%	3.3%	2	9.5%	4.7%
Overdrainage		10	20.0%	10.9%	3	14.3%	7.0%
Infection		8	16.0%	8.7%	8	38.1%	18.6%
Unspecified		3	6.0%	3.3%	0	0%	0%

### Follow-up

For all 92 patients, follow-up time was determined from the initial VPS surgery to the first revision, death, or September 30, 2024. The follow-up period was from January 1, 2003, to September 30, 2024. The mean follow-up time for all VPS patients (*n* = 92) was 5.9 years (SD 6.8 years), and the mean follow-up time for shunted patients with the Strata valve (*n* = 43) was 5.3 years (SD 5.3 years). One patient was lost to follow-up as a result of moving to a different catchment area.

### Shunt valve and catheter types

During the study period, eight types of valves, both adjustable pressure and fixed-pressure valves, were used in patients requiring a shunt for CSF diversion (Table 2). Since 2009, only Strata adjustable valves have been used in our institution. The majority of the valves used at KUH were adjustable pressure valves (*n* = 58; 63%), with the Strata adjustable valve (Medtronic, Minneapolis, MN, USA) being the most commonly used (*n* = 43; 46.7%), both with and without an antisiphon part (Strata II adjustable and Strata NSC adjustable). The Strata II valve with an integrated Delta chamber was used in 37 patients, whereas the Strata NSC valve without a Delta chamber was used in six patients. In this study, Strata adjustable valves were also studied in a separate group. Other commonly used valve types included the fixed-pressure Medtronic 1.5 (*n* = 21; 22.8%) and the adjustable Codman Hakim (Codman Neuro, Raynham, MA, USA) (*n* = 15; 16.3%). All other shunt valve types are summarized in Table 2. There was no statistically significant difference in the risk of revision between valve types (*P* = 0.362), or whether the primary valve was non-adjustable or adjustable (*P* = 0.821). Furthermore, no statistically significant difference in the risk of shunt revision was observed when comparing Strata adjustable valves with Codman adjustable valves (*P* = 0.233) or non-adjustable valves (*P* = 0.539).

During the study period, three different types of ventricular shunt catheters were used: silicone (*n* = 67; 72.8%), antibiotic-impregnated (*n* = 23; 25%), and Silverline (*n* = 2; 2.2%). Catheter type did not show a statistically significant difference regarding shunt revision (*P* = 0.906). Most of the ventricular catheters were placed occipitally (*n* = 85; 92.4%), and the rest frontally (*n* = 7; 7.6%).

### Revision rate of shunts

In our data, the shunt revision rate was 54.3% (*n* = 50) during the follow-up period (Table 2 and Fig. 2A). The mean time from the primary shunt operation to the first revision was 1.0 year (SD 1.8 years). In 58% (*n* = 29) of the patients who required revision surgery, more than one revision was necessary. Most of the revisions were done within 1 year after the initial shunt surgery (*n* = 33; 66%), resulting in a 1-year revision rate of 35.9%. Underdrainage was the leading cause for shunt revision in 58% (*n* = 29) of cases, overdrainage in 20% (*n* = 10) of cases, and infection in 16% (*n* = 8) of cases.

The revision rate for primary shunts with the Strata valve was slightly lower (*n* = 21; 48.8%), with the mean time from primary surgery to the first revision being 1.0 years (SD 1.8 years). More than half (*n* = 13; 61.9%) of the patients who required revision underwent more than one revision surgery in this group. Most of the revisions were done within 1 year after the initial shunt surgery (*n* = 15; 71.4%), resulting in a 1-year revision rate of 34.9%. Underdrainage was the most common reason for revision (*n* = 10; 47.6%), followed by infection (*n* = 8; 38.1%) and overdrainage (*n* = 3; 14.3%). All causes for shunt revisions are summarized in Table 3.

### Risk factors for shunt revision

Gender (*P* = 0.821), birth weight (*P* = 0.395), birth weight under 1500 g (*P* = 0.299), etiology of HC (*P* = 0.118), prior use of a Rickham capsule (*P* = 0.418), prior use of an EVD (*P* = 0.349), infection within 2 months before the initial shunt surgery (*P* = 0.510), catheter type (*P* = 0.906), ventricular catheter placement site (*P* = 0.415), shunt valve type (*P* = 0.362), adjustable pressure valve adjustments (*P* = 0.324), and primary adjustable valve (*P* = 0.821) were not statistically significantly associated with an increased risk of shunt revision. Patients younger than 6 months had a higher risk of shunt revision compared to older patients (*P* = 0.036). When comparing the CD (*P* = 0.044) and IVH (*P* = 0.165) etiology groups with the tumor group, a statistically significant association with higher revision rates could be demonstrated in the CD group. Categorical variables were analyzed using Pearson’s chi-squared test or Fisher’s exact test, and continuous variables using independent samples *t*-test.

The log-rank test comparing patients under 6 months old at the initial surgery to the rest of the study population showed a statistically significant difference in shunt survival (*P* = 0.030, log-rank test). When comparing the most common etiologies (tumor, IVH, and CD), a trend of shunt survival being worse in the CD and IVH groups compared to the tumor group was observed, but the difference was not statistically significant (*P* = 0.160, log-rank test). Among patients with adjustable pressure shunt valves who underwent at least one valve adjustment, there was a trend toward reduced shunt survival, although this difference did not reach statistical significance (*P* = 0.407, log-rank test). Comparison of revision-free shunt survival between Strata adjustable valves and other valve types revealed no statistically significant difference (*P* = 0.438, log-rank test). Kaplan–Meier survival functions are presented in Fig. 2*.*

In a univariable Cox regression, age younger than 6 months at primary operation was associated with a higher risk of shunt revision (*P* = 0.038, HR 2.4, CI 1.05–5.65). Other variables showed no statistically significant association with shunt revision in either univariable Cox regression or logistic regression analyses. Multivariate logistic regression and Cox regression analyses were performed on variables associated with shunt revision, but no statistically significant associations were found.

### Causes of death

During our study period from 2003 to 2024, there were 22 (23.9%) deaths, of which 17 were not related to HC or CSF diversion surgery. In five patients, the exact cause of death remained unknown. Most deaths were tumor-related (*n* = 12), including medulloblastoma (*n* = 3), glioma (*n* = 2), oligoastrocytoma (*n* = 2), pilocytic astrocytoma (*n* = 2), PNET (*n* = 2), and retinoblastoma (*n* = 1). CD-related deaths (*n* = 4) were also observed, including encephalocele (*n* = 1), schizencephaly (*n* = 1), cloverleaf skull syndrome (*n* = 1), and myelomeningocele (*n* = 1). One child died of acute myeloid leukemia.

## Discussion

This retrospective, single-center, population-based study included 92 pediatric patients who required primary CSF diversion surgery due to hydrocephalus over a 17-year period. In total, 35.9% of shunted patients required revision surgery within 1 year, and 54.3% required shunt revision during the follow-up. The most common reasons for revision surgery were overdrainage and ventricular catheter obstruction, followed by infection. We found that age under 6 months and congenital defects as the etiology increased the risk for shunt revision.

Regarding the age, we found that patients who were less than 6 months old at the time of initial shunt placement were at a higher risk for shunt revision surgery compared to older patients (*P* = 0.036). Similar findings have been observed in several previous studies, where age has been a major determinant of the outcome of CSF diversion procedures, either shunt or ETV, with worse outcomes in younger patients [12, 14, 22, 28, 29, 36]. Moreover, age under 6 months or less at the time of the first shunt surgery predicts a higher risk for shunt revision [4, 30, 32, 35, 37] or revision after endoscopic surgery [5, 14].

Certain etiologies, such as congenital HC and IVH, have been previously shown to be associated with an increased risk for shunt revision [22, 26, 29, 36]. In this study, a comparison of shunt survival among patients with different etiologies revealed higher revision rates for HC caused by congenital defects compared to patients with tumor-related HC (*P* = 0.044), which is in line with our previous study [36]. However, in the current study, which included both a larger study population and longer follow-up, we did not find a significant difference in shunt revision rate between the IVH and tumor groups. The difference in the findings may be explained by tumor-related deaths and the longer mean follow-up in the present study.

The infection rate for primary shunts was 8.7%, which is comparable to the results of previous studies with infection rates between 4 and 15% [3, 13, 27, 30, 33, 39]. Factors such as age under 6 months and MCC have been associated with an increased risk of shunt infection, whereas the neoplastic etiology of HC has been found to be a protective factor against infection [13, 22, 27, 33]. Even though antibiotic-impregnated catheters have been previously shown to reduce the risk of shunt infections [21, 35], we found no difference in shunt revision risk with different types of ventricular catheters (*P* = 0.906). It is likely that the use of antibiotic-impregnated catheters shifts the cause of shunt revision from infection to mechanical malfunction. The insertion of a Rickham capsule (*P* = 0.418) or EVD (*P* = 0.349) prior to primary shunt surgery did not increase the risk of shunt revision or infection in our study population. Patients with hemorrhage-related HC mainly received Rickham capsules, while EVD was mostly used in patients with tumor-related HC. In a previous study comprising preterm infants with HC caused by IVH, no significant difference in shunt revision rates was found when patients with ventricular reservoirs prior to shunt placement were compared with patients who were shunted initially [7]. However, in a retrospective cohort study with a population size comparable to our study, prior EVD placement was associated with early 30-day shunt failure, but this association was no longer evident at the 6-month follow-up [26].

Despite extensive research during the last decades, no superior valve type has been found. The objective of our shunt treatment policy has been to avoid overdrainage; thus, the majority of shunted patients in our study received an adjustable pressure valve (63%), of which 74.1% were Strata valves. We found a slight trend toward better shunt survival with the Strata adjustable valve since the revision rate for this group was 48.8%, which is slightly lower than the overall revision rate of 54.3% for all shunted patients. However, since the Strata valves were mainly placed in the latter two-thirds of the study period, their mean follow-up time was slightly shorter (5.3 years vs. 5.9 years for all shunts). No statistically significant difference in revision rates was found between Strata valves and other valve types (*P* = 0.392), nor between adjustable pressure and fixed-pressure valves (*P* = 0.821). Within the Strata valve group, no patient variable achieved a significant difference for the risk of shunt revision, which is likely attributed to the smaller sample size (*n* = 43) and shorter follow-up period of this group. The existing literature contains a certain degree of controversy regarding the impact of valve type on shunt survival. Previously published meta-analyses indicate that adjustable valves are less prone to over- or underdrainage complications and shunt revision compared to non-adjustable valves [20, 43]. A recent single-center study suggests that the use of differential pressure shunt valves may result in longer shunt survival compared to gravitational valves but found no significant difference between adjustable and non-adjustable valves [41]. However, in a 1998 published randomized trial with a large pediatric population, Drake et al. [11] compared three different shunt valve types without finding a difference in shunt survival. Furthermore, in more recent studies, no significant difference in shunt survival was observed between adjustable and non-adjustable valves [1, 15, 30]. Thomale et al. [39] and Gebert et al. [3] reported results comparable to ours when using adjustable differential pressure valves with a gravitational unit (proGAV). Both Gebert’s [3] infant cohort and Thomale’s [39] broader pediatric series demonstrated that gravity-assisted adjustable valves are durable, yet most revisions were driven by catheter-related problems rather than valve failure. Outcomes were consistently poorer in infants, consistent with our finding that children younger than 6 months have a higher risk of shunt revision, with most revisions clustering in the first postoperative year and a substantial burden persisting long term. Thus, while gravity-assisted adjustability may help limit valve-related complications, it does not overcome the predominant risk of catheter malfunction.

A review of the literature identified nine relevant shunt cohorts with a mean follow-up period of at least 1 year, ranging up to 19 years (Fig. 1 and Table 1). The studies that analyzed shunt survival for 1 year had lower revision rates (18% to 33%) [2, 4, 28], whereas the studies that had longer follow-up times reported higher revision rates (47.5% to 72.8%) [6, 19, 22, 31, 36, 41]. In some of these studies, the comprehensive follow-up was not achieved, affecting most probably the revision rate [4, 28]. Our 1-year shunt revision rate of 35.6% and long-term revision rate of 53.5% are consistent with these findings. Moreover, the shunt revision rate remained consistent (54.3% vs. 51%) compared to our previous publication, despite an increased study population and extended follow-up [36]. The mean time to the first shunt revision in our study was 1.0 years, which is consistent with existing literature reporting a range from 3 months to 2 years [19, 22, 34, 36]. Our 6-year mean follow-up period was the third longest among the relevant studies. KUH is a referral center with a large population base, which enables a comprehensive long-term follow-up on patients treated in our catchment area, and therefore, very few patients were lost during the follow-up. The most common HC etiologies in the above-mentioned relevant cohorts, including tumors, congenital defects, aqueductal stenosis, and hemorrhage-related causes, are comparable with our findings [2, 6, 19, 22, 28, 31, 36, 41]. Younger age, particularly under 6 months at the time of the initial shunt surgery, and HC etiologies such as congenital HC, IVH, and MMC have been found to be associated with a higher risk for shunt revision [4, 22, 36]. In a recent study, the use of gravitational shunt valves compared to differential pressure valves was associated with a higher risk of shunt revision, but the previous randomized trial did not confirm this finding [11, 41].

The present study is subject to inherent limitations of a retrospective study design and a relatively modest sample size. As with any retrospective study, there is a risk of bias, including selection bias and the influence of confounding factors, which may obscure the true nature of the relationships between variables. However, the strengths of our study include its extended follow-up period and population-based approach, providing a thorough examination of risk factors for revision surgery in pediatric HC patients. Importantly, only one patient was lost to follow-up during the study period. We acknowledge that further studies with larger pediatric patient cohorts or multi-center studies are necessary to confirm our findings and to make meaningful interpretations regarding risk factors for shunt revision.

## Conclusions

In this study, the long-term revision rates for primary CSF diversion surgery in pediatric patients were 54.3% (mean follow-up 5.9 years) for all shunted patients and 48.8% (mean follow-up 5.3 years) in the Strata adjustable valve group. The 1-year shunt revision rate was 35.9%, with the majority of revisions (66%) occurring within the first year. Younger age, particularly age under 6 months old at the initial shunt surgery, was associated with poorer shunt survival. Shunted patients with HC caused by congenital defects had a higher risk for shunt revision. The use of the Strata adjustable valve versus other valve types did not significantly affect shunt survival. Due to the limited study population, no significant factors associated with revision surgery could be identified in the Strata adjustable valve group.

## Data Availability

No datasets were generated or analysed during the current study.
